# Gamification as a tool to teach key concepts in microbiology to bachelor-level students in biology: a case study using microbial interactions and soil functioning

**DOI:** 10.1099/acmi.0.000699.v3

**Published:** 2024-02-06

**Authors:** Aislinn Estoppey, Camille Tinguely, Melissa Cravero, Estelle Blandenier, Lucie Brand, Julien Court, Marie Zaninetti, Saskia Bindschedler, Pilar Junier

**Affiliations:** ^1^​ Laboratory of Microbiology, University of Neuchâtel, Rue Emile-Argand 11, CH-2000, Neuchâtel, Switzerland

**Keywords:** gamification, soil functioning, litter degradation, bacteria, fungi, archaea

## Abstract

Microbiology is a difficult topic to teach given that the objects of study are mostly invisible to the learner. The majority of university students beginning their training in biology are more interested in natural objects that can be seen with the naked eye. Nonetheless, micro-organisms are key components of the biosphere and a good microbiological background is required for a thorough training in natural sciences. Lectures are still a common teaching format in universities. However, it is a passive learning format and no longer considered the most adequate approach in most teaching situations. Instead, alternatives consisting of more active teaching formats have been recognized to better motivate students to acquire and consolidate knowledge. In addition, transferable skills, such as effective communication, critical thinking and time management, are acquired simultaneously. A similar engagement can be obtained using games as part of the teaching experience. In this study, we designed a card game to teach key concepts in basic bacteriology and mycology to bachelor-level students. The first task consists of creating and designing microbial characters based on a list of species. This proved very useful for second-year bachelor students in terms of grasping concepts such as cell morphologies, taxonomy and life cycles. In the second task, third-year students used the characters created in the second-year class to develop a game based on an ecological function, namely forest litter degradation. In addition, they also considered experimental validation of the microbial activities and incorporated knowledge acquired in other fields.

## Impact statement

Micro-organisms are key components of the biosphere and their diversified metabolisms can be at the centre of concrete sustainable solutions to tackle future societal challenges, such as the climate and resource crises. Therefore, a solid microbiological background is required for a thorough training in natural sciences. Microbiology is a difficult topic to teach given that the objects of study are for the most part invisible to the learner. As a result, innovative teaching methods are needed to increase students’ interest in the invisible world. Enhanced engagement can be obtained by the use of games as part of the teaching experience. Games are already used for microbiology teaching, but most perpetuate the association of micro-organisms with their negative facets (e.g., diseases). In contrast, we designed a new card game aimed at teaching fundamental concepts in basic bacteriology and mycology to bachelor-level students, tapping in on the positive impacts of microbial life on ecosystem functioning. The game can be used at different levels to teach basic (e.g., morphology, life cycles and taxonomy) or advanced (e.g., metabolism, ecosystem function and biological interactions) concepts in microbiology.

## Data Summary

The authors confirm all supporting data and protocols have been provided within the article or as supplementary data files.

## Introduction

Curricula in biology typically involve the teaching of content-rich topics, which are prone to relying on memorization rather than on learning by comprehension. This model is enhanced by a teaching format that is centred on a teacher providing theoretical content without any interaction between the teacher and the students, or between students. The result is often uninspiring or ineffective lessons. When students are actively involved in the learning process, they can go beyond memorization and achieve a higher level of understanding and retention. This can be done using problem-solving activities, active research or group work [1]. The integration of games in education is currently widely acknowledged as a tool that improves learning outcome and fosters students’ engagement. Various categories of games can be incorporated into teaching strategies. These range from the inclusion of so-called ‘serious games’ (i.e., games with a function of learning rather than entertainment), ‘learning games’ (i.e., games with a clear didactic objective used to support learning) or ‘gamification’, which consists of the use of elements typical to games (e.g., challenges and level points) within the context of teaching [2]. There is a large body of research on the effect of games on teaching, many of which focus on the use of digital tools [3]. Multiple beneficial aspects can be attributed to the use of gaming in biology teaching, including accessibility, applicability and flexibility, improved motivation, engagement and learning, as well as acting as a trigger for the development of new skills [4].

Microbiology presents a unique set of challenges and opportunities regarding teaching. An important challenge is that the objects of study are for the most part invisible to the learner, making the teaching of basic concepts extremely abstract. In addition, in many universities, microbiology is only one topic in a larger curriculum that includes other topics in natural sciences, and it is often not the primary motivation of the students to engage in a career in biology. The use of games as part of the teaching of microbiology could improve the learning and teaching experience for both students and trainers. When games are considered as part of the toolkit for teaching microbiology, these challenges represent opportunities, as invisible objects can become visible in a playful context. However, one point that is often reinforced by the available gaming tools in microbiology is the link between micro-organisms and infectious diseases [5–8], often concentrating solely on the role of micro-organisms in human health [9]. In contrast, there are many positive aspects of microbiology that are entirely neglected; including for instance, the provision of important ecosystem services.

The aim of this project was to develop a game to facilitate the learning of basic concepts in bacteriology (including the comparison with archaea) and mycology, and to consolidate those concepts using a playful and stimulating learning process. A second goal was to concentrate on the positive aspects of microbiology to develop the game’s dynamics.

## Methods

### Target population and organization of the activities

**Fig. 1.**
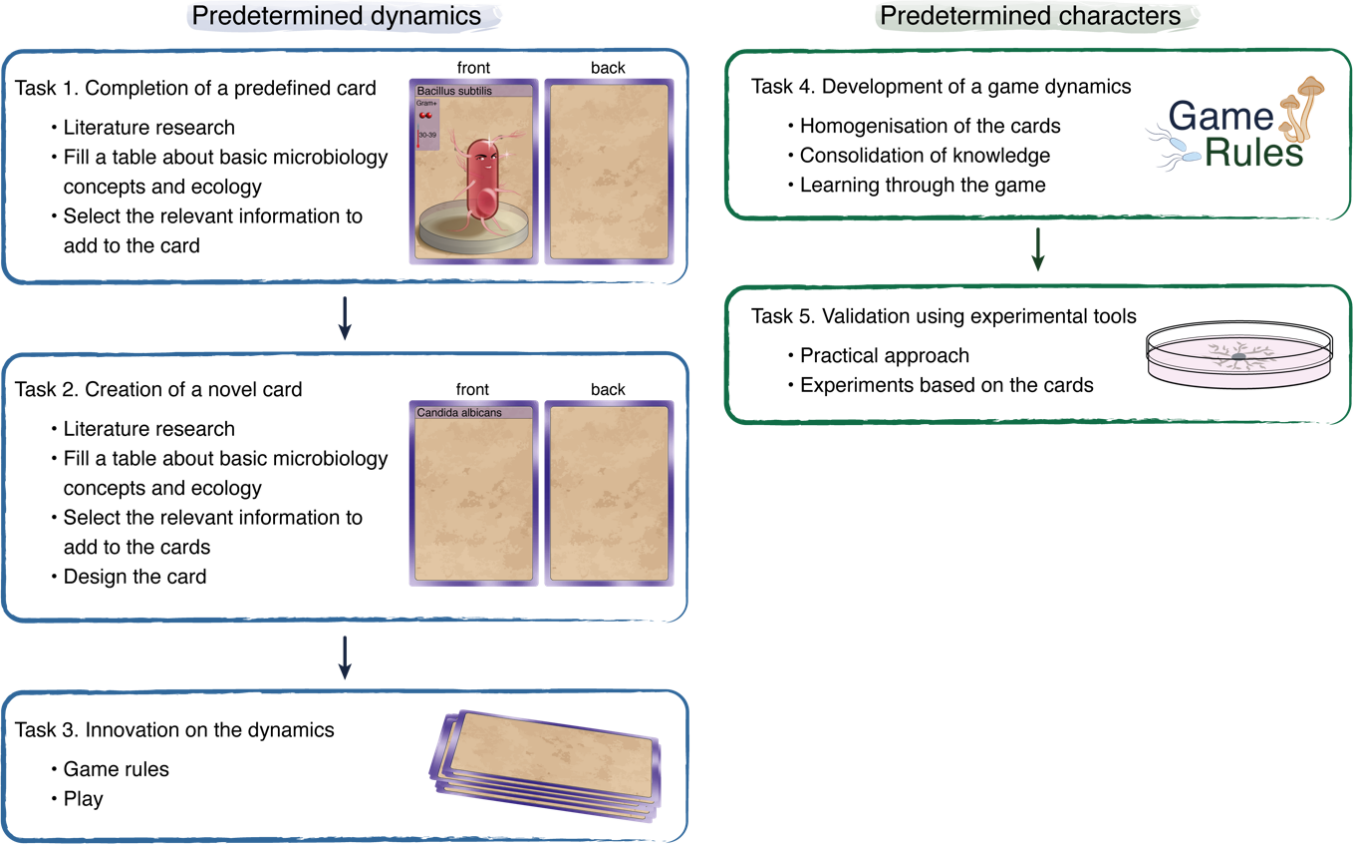
Tasks for the development of the game. Second-year students were assigned tasks 1–3 (left), while third-year students completed tasks 4–5 (right).

The target population was second- and third-year biology undergraduate students (bachelor level). They were engaged in a semester-long programme (second-year students) and a 4-week problem-based immersive module (third-year students). The game was developed with the active participation of the students in two steps: the first consisted of the development of character cards for predetermined game dynamics, while the second step focused the development of game dynamics using predetermined characters (Fig. 1). For the second-year students, the focus was learning basic vocabulary and understanding the general features of the studied microbes. For this, they designed predetermined cards, created novel cards and played with the basic game dynamics (Tasks 1–3 in Fig. 1). The third-year students used the cards created by the second-year students to further develop the game dynamics by using the role of micro-organisms in the degradation of the organic matter of temperate forest soil as the guiding activity. Moreover, they were tasked with validating theoretical knowledge through an experimental approach (Tasks 4–5 in Fig. 1).

### Card development

**Fig. 2.**
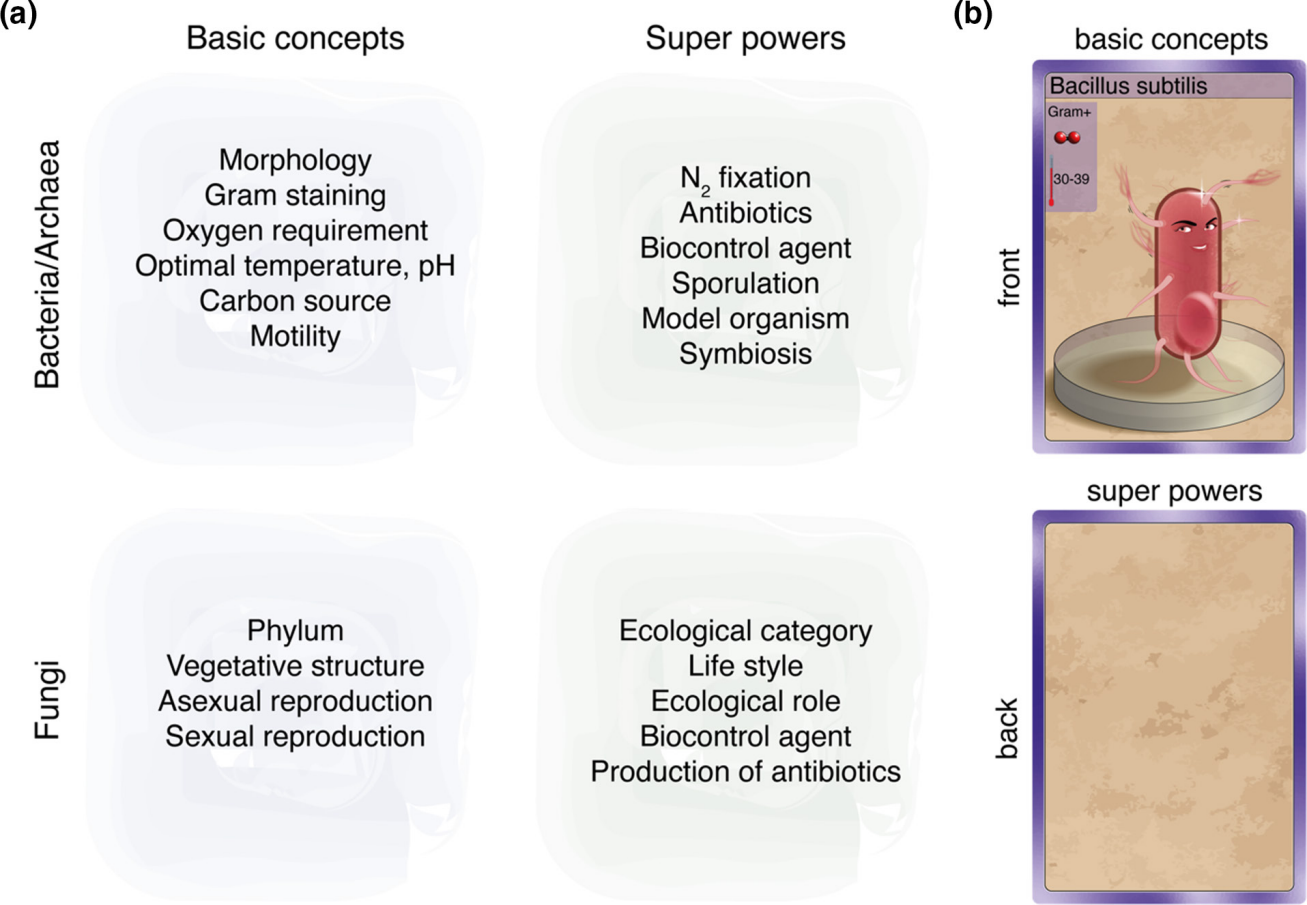
Concept and card examples. A list of key concepts that are evaluated at the end of the lectures in bacteriology and mycology was prepared (for details please see Supplementary Data 1). These were separated between basic concepts and ‘super-powers’ (**a**). For half of the cards, the card design for the face was done beforehand, while the back was left blank (**b**).

For the purpose of the game, cards were designed with a visual representation of the character (bacterium or fungus) supplemented by basic concepts and so-called ‘superpowers’ related to their metabolism and ecology (Fig. 2a; Supplementary Data 1, available in the online version of this article). The design of the front/face of the cards was done beforehand for half of the characters, while the backs were left blank (Fig. 2b). These cards served as models for the development of the other cards. A total of 18 bacteria and 16 fungi (Table 1) were selected based on the content covered in the theoretical bacteriology and mycology courses. The selection was made to facilitate the learning of the different concepts depicted in Fig. 2a.

**Table 1.** List of the pre-selected micro-organisms (i.e., characters) and rationale for their selection

Bacteria	Rationale
*Ammoniphilus oxalaticus*	Obligate oxalotroph, endospore-forming bacterium
*Anabaena azollae*	Cyanobacterium, nitrogen fixation
*Bacillus subtilis*	Model Gram-positive endospore-forming bacterium
*Borrelia* spp.	Spirochete, endoflagella; Lyme disease causing agent
*Chlamydia* spp.	Intracellular parasite, antibiotic resistance
*Clostridium* spp.	Anaerobe, potential pathogen
*Cupriavidus necator*	Use in biotechnology
*Helicobacter pylori*	Human pathogen
*Heliobacterium* spp.	Anoxygenic photoheterotrophy
*Mycoavidus cysteinexigens*	Fungal endosymbiont
*Myxoccocus* spp.	Myxospores producer; cooperative behaviour
*Pseudomonas protegens*	Plant-protecting bacterium
*Pseudomonas putida*	Model Gram-negative organism, use in biotechnology
*Rhizobium* spp.	Plant symbiont, nitrogen fixation
*Rhodobacter* spp.	Photoautotrophy, photolithotrophy, nitrogen fixation
*Streptomyces* spp.	Filamentous growth, antibiotic production
*Wolbachia* spp.	Insect endosymbiont, intracellular parasite
**Fungi**	**Rationale**
*Aspergillus niger*	Black mould, oxalic acid production
*Batrachochytrium dendrobatidis*	Amphibian pathogen
*Beauveria bassiana*	Saprophyte, entomopathogen, biocontrol agent
*Candida albicans*	Human microbiota, opportunistic pathogen
*Coprinopsis cinerea*	Saprophyte, coprophagous
*Entomophthora muscae*	Entomopathogen
*Fusarium oxysporum*	Saprophyte, symbiont, phytopathogen
*Malassezia* spp.	Human microbiota, opportunistic pathogen, saprophyte
*Morchella crassipes*	Ectomycorrhiza, edible
*Neocallimastix* spp.	Symbiont, rumen
*Paxillus involutus*	Symbiont, ectomycorrhiza
*Rhizophagus irregularis*	Endomycorrhiza
*Rhizopus oligosporus*	Saprophyte, use in biotechnology
*Trametes versicolor*	White rot, dead wood decomposition
*Trichoderma viride*	Green rot, saprophyte, symbiont, parasite
*Ustilago maydis*	Corn smut, saprophyte, parasite

### Session plan: second-year

For creation and completion of the cards, groups of 3–4 students used literature research (approximately 1–1.5 h of activity per card). Each student group was responsible for conducting literature research on four micro-organisms (two bacteria and two fungi). In addition, the students were assigned the task of developing a card for an archaeon, with the freedom to choose the specific character. The literature review allowed them to complete the pre-existing cards and to design their own for the new characters. Also, with the help of the literature, each group selected relevant information for their characters. The content of the cards was validated during lectures in which specific sessions (approximately 2 h of work) were planned for the presentation and discussion of the cards, allowing them to improve the content and design. Once the cards were completed, students were encouraged to play for approximately 3 h at the end of the semester.

### Pre-defined game dynamics

For the gaming sessions, two types of approaches were tested. In the first one, the students were presented with pre-defined game dynamics. The game corresponded to a placing game – ‘Party invite: place the characters in different rooms, based on their morphological, ecological or application properties’ (Table 2) – in which the goal was to use the content of the cards and the supporting table to correctly identify the categories (rooms) in which a given microbial character belongs. For this, groups of 4–6 students were given a card deck containing characters and rooms. Each participant was given 4–5 cards, and the ‘room’ card was placed in the centre. In turns, based on their own knowledge, each participant places one card corresponding to that category. With the help of the table, the players confirm if the placement is correct. If the placement is correct, the next player takes their turn to play. If the player has made a mistake, they have to take back the card and take a penalty card from the remaining cards in the deck. After each turn, a new room is placed in the centre. The goal of the game is to be the fastest to play all the cards in their hand (i.e., no cards remaining in hand).

**Table 2.** Party invite pre-defined game dynamics

Room	Category	Rationale
Dimorphism	Morphology	Organisms with multiple cellular forms or morphologies during their life cycle
Dressed in violet		Bacteria with a Gram-positive staining
Dressed in pink		Bacteria with a Gram-negative staining
Dead food	Ecology	Organisms with at least a partial ability to grow based on the degradation of dead organic matter (saprotrophic growth)
Happy couple		Organisms known to establish a mutually beneficial interaction with another micro-organism or a macro-organism
Manipulating your host		Organisms with the ability to manipulate the behaviour or physiology of a host
Pathogens club		Organisms with a known pathogenic lifestyle at least in part of their life cycle
Stress resistance		Organisms known for the production of a cell type allowing them to withstand environmental stress
Insect killer		Known entomopathogenic organisms
Keeping fungi at bay	Application	Organisms that are known to be antagonistic to fungi
Helping plants		Organisms with known plant-growth promoting traits
Biotech geeks		Organisms known for their use in biotechnology
All allowed	General	Any cards can be placed in this room.

Description of the rooms and criteria allowing participants to place the microbial characters on the cards in a specific room. Four categories were considered for the selection of the rooms: morphology, ecology, application and general.

From the second gaming session, the students were encouraged to develop new gaming rules to learn and reinforce the understanding of the concepts presented during the lectures in a playful manner. The students then submitted the rules to the trainers when they considered the game dynamics stable.

### Session plan: third-year

The third-year students were presented with the collection of cards prepared by the second-year students and asked to create a game based on the cards. This activity spanned 4 weeks as part of an immersive module within a problem-based learning framework, taking place in the microbiology laboratory. The students selected forest litter degradation as the ecological function for the development of a cooperative game (activity accomplished in the first week). Based on that ecological function, the students selected the cards corresponding to the most relevant microbial characters and focused on their degradation capabilities. The backs of the cards were homogenized to indicate key features related to the game dynamics (activities accomplished the second and third week). Once the game was developed, the students invited fellow third-year students to play the game and to provide feedback on the dynamics and the importance of the game for learning microbiology (fourth week).

### Experimental validation of degradation activities

The third-year students were also given the task of proposing experiments to evaluate the predictions made during the design of the game. Out of the 34 cards, 12 micro-organisms were evaluated based on their ecology and availability in the bacterial and fungal culture collections of the Laboratory of Microbiology of the University of Neuchâtel (Table 3). The selected organisms were grown aerobically at 22 °C (fungi) or 30 °C (bacteria) on potato dextrose agar (PDA; 39 g l^−1^; Carl Roth) for the initial inoculum. Experiments were run to determine the capacity of the micro-organisms to degrade starch, pectin, lignin, cellulose or organic nitrogen by growing them on media containing the polymer of interest (see the sections below and a summary in Supplementary Data 2). In addition, the capability to fix nitrogen was also evaluated. Experiments were done in triplicate and results were collected in a qualitative manner (yes/no answers). For the inoculation of the bacterial strains, from an overnight culture on PDA, a colony was picked and reinoculated in the selected medium using a streaking out approach. The plates were sealed with parafilm and incubated at 30 °C until clear microbial growth. For the inoculation of fungi, a piece of actively growing mycelium was cut from the margin of a colony grown in PDA. The piece was then transferred to fresh medium. The plates were sealed with parafilm and incubated at 22 °C until clear microbial growth. The same procedure was used for the inoculation of liquid media, but those were incubated at 30 °C (bacteria) or 22 °C (fungi) under agitation (108 r.p.m.).

**Table 3.** List of the micro-organisms used for the experimental validation

Bacteria	Strain code
*Bacillus subtilis*	NEU1*
*Cupriavidus necator*	DSM4182
*Pseudomonas protegens*	CHAO
*Pseudomonas putida*	DSM291
*Streptomyces avermitilis*	DSM46492
**Fungi**	**Strain code**
*Aspergillus niger*	A1144
*Beauveria bassiana*	KVL03-125
*Coprinopsis cinerea*	M141*
*Fusarium oxysporum*	M195*
*Morchella crassipes*	M21-48*
*Trametes versicolor*	M130*
*Trichoderma viride*	M136*

*Code corresponds to the identifier used in our culture collection.

#### Starch degradation

The micro-organisms were inoculated on starch agar medium (K_2_HPO_4_, 0.2 g l^−1^; NaCl, 0.1 g l^−1^; SL6 solution 1 ml l^−1^ – ZnSO_4_·7H_2_O, 100 mg l^−1^; MnCl_2_·4H_2_O, 30 mg l^−1^; H_3_BO_3_, 300 mg l^−1^; CoCl_2_·6H_2_O, 200 mg l^−1^; CuCl_2_·2H_2_O, 10 mg l^−1^; NiCl_2_·6H_2_O, 20 mg l^−1^; Na_2_MoO_4_·2H_2_O, 30 mg l^−1^ –; soluble starch, 1.5 g l^−1^; NH_4_NO_3_, 1 g l^−1^; agar, 15 g l^−1^) in which starch was the sole carbon source. When growth was visible, Lugol’s iodine staining solution (I_2_, 5 g l^−1^; KI, 10 g l^−1^) was deposited directly onto the agar plate to reveal degraded (brownish to transparent) *versus* non-degraded starch (dark blue).

#### Pectin degradation

Micro-organisms were inoculated on an agar medium in which pectin was the sole carbon source. The medium was composed of one part of solution I and one part of solution II. Solution I contained 5 g l^−1^ CaCl_2_ and 15 g l^−1^ agar. Solution II (pH 8) contained 0.05 g l^−1^ K_2_HPO_4_, 0.15 g l^−1^ KH_2_PO_4_, 0.2 g l^−1^ MgSO_4_·7H2O, 0.002 g l^−1^ Na_2_MoO_4_·2H_2_O, 1 g l^−1^ NH_4_NO_3_, 0.01 g l^−1^ FeCl_3_. Solution II was mixed with 16 g l^−1^ ethanol-sterilized pectin after autoclaving. The amount of agar in solution I is not sufficient for jellification and pectin acts as a gelling agent. Therefore, its degradation is made visible by the presence of liquid droplets and/or transparent halos around fungal hyphae and bacterial colonies.

#### Lignin degradation

Micro-organisms were inoculated on Remazol Brilliant Blue R medium (NaCl, 1 g l^−1^; yeast extract 0.1 g l^−1^; MES, 1.95 g l^−1^; Remazol Brilliant Blue R sterilized by filtration at 0.22 µm, 0.5 g l^−1^; agar, 20 g l^−1^, pH 5), which has an analogous chemical structure to lignin [10]. A discolouration of the dark blue medium indicated the organism’s ability to degrade lignin [11].

#### Cellulose degradation

Micro-organisms were cultivated in carbon-free liquid medium (K_2_HPO_4_, 0.2 g l^−1^; NaCl, 0.1 g l^−1^; SL6 solution, 1 ml l^−1^; NH_4_NO_3_, 1 g l^−1^), in which cellulose fibres (cigarette rolling papers) were added. When growth was visible, cellulose papers were removed from the cultures and stained with 0.1 % Congo red (Fluka) for 5 min before being washed with 1 M NaCl. The absence of coloration indicated the degradation of cellulose [12].

#### Organic nitrogen degradation

Micro-organisms were inoculated on skimmed milk agar medium (skimmed milk, 10 g l^−1^; malt extract, 12 g l^−1^; agar, 15 g l^−1^) in which casein was the sole nitrogen source (modified from DSMZ Medium 170). The culture medium is cloudy and the apparition of a transparent halo around the colonies indicates the degradation of organic nitrogen [13].

#### Atmospheric nitrogen fixation

Micro-organisms were cultivated in nitrogen-free solid medium (glucose, 20 g l^−1^; K_2_HPO_4_, 0.05 g l^−1^; KH_2_PO_4_, 0.15 g l^−1^; CaCl_2_, 0.01 g l^−1^; MgSO_4_·7H_2_O, 0.20 g l^−1^; Na_2_MoO_4_·2H_2_O, 0.002 g l^−1^; FeCl_3_, 0.01 g l^−1^; CaCO_3_, 1 g l^−1^; agar, 15 g l^−1^). As a control, the micro-organisms were grown in the same medium supplemented with ammonium nitrate (NH_3_NO_4_, 1 g l^−1^). Organisms that were able to grow in nitrogen-free medium were considered to have the ability to fix atmospheric nitrogen.

## Results

### Completion of the cards

Groups of 3–4 participants were created among the second-year students. Each group was assigned a list of four microbes among the 34 pre-selected organisms.

**Fig. 3.**
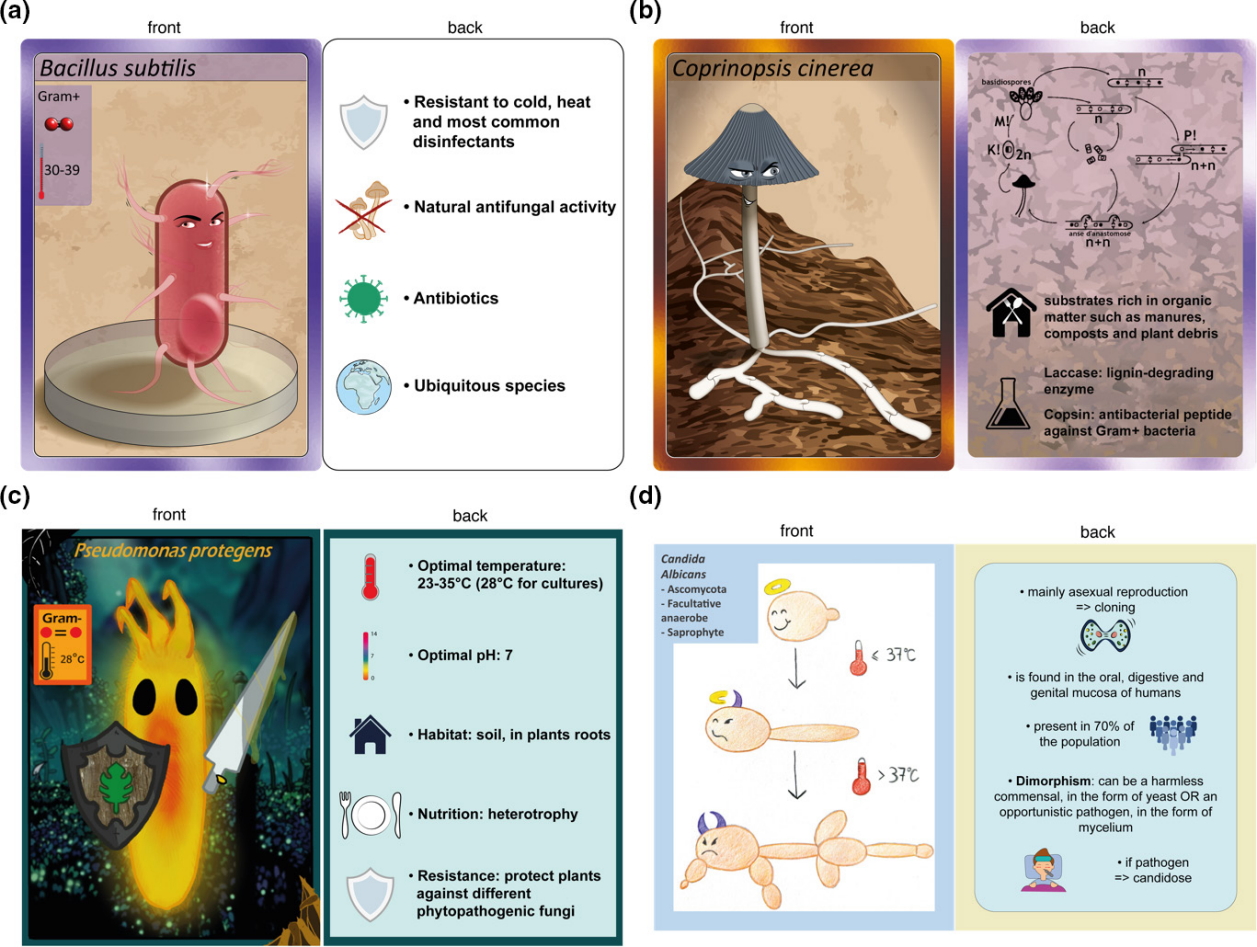
Examples of cards designed by the second-year students with a pre-defined design for the face (**a–b**) or without a model (**c–d**) for bacteria (**a–c**) and fungi (**b–d**).

To guide the literature research, the groups were encouraged to fill in a table with relevant information on the metabolism and the ecology of each micro-organism (Supplementary Data 3 and 4). This was done to assist the students in the analysis of literature. Also, to help them with the card design, for the first set of cards, a model of the illustration was provided (Supplementary Data 5). Based on their findings, the students selected the key elements to be represented on the each character card (Fig. 3a, b) and developed an iconography system to represent those elements (Fig. 3c–f). The choice of key elements was highly variable between the groups, but some characteristics, such as the optimal temperature, Gram-staining, oxygen requirement, optimal pH and morphology, were found on most of the cards for bacteria (Table 4). For fungi, students focused on cell shape (unicellular), macroscopic form of the fruiting bodies, ecological category, taxonomy (phylum), habitat and reproduction, while characteristics such as temperature and pH were only found on a few cards (Table 4). Important features, such as the formation of endospores (Fig. 3a), the formation of clamp connections (Fig. 3b), intrinsic fluorescence (Fig. 3c) or the dimorphic phenotype in fungi (Fig. 3d), were also highlighted on the face side of the cards as part of the character illustrations. Each character was also given superpowers, which ranged from the fixation of atmospheric nitrogen to their use as biocontrol agents or in biotechnology. The accuracy of the content of the cards was discussed during presentations made in class based on the information provided in the accompanying tables (Supplementary Data 3 and 4).

**Table 4.** Features presented on the cards by the students and the number of cards presenting a given feature

Bacteria	Number of cards
Optimal growth temperature	24
Gram-staining	23
Oxygen requirements	20
Optimal pH	17
Shape (e.g., bacilli, cocci)	16
Nutrition (e.g., autotroph, heterotroph)	16
Habitat	14
Life cycle	12
Motility	11
Super power	10
Production of spores	7
Resistance	6
Taxonomy	4
Production of antibiotics	2
**Fungi**	**Number of cards**
Shape	25
Ecological category	24
Phylum	22
Habitat	22
Reproduction	20
Super powers	16
Optimal growth temperature	13
Ecological role	10
Life cycle	4
Biotechnology	4
Optimal pH	3
Oxygen requirements	2

The total number of cards corresponded to 36 bacteria and 32 fungi.

In addition to the cards for bacteria and fungi created and discussed in class, the students were given the choice to prepare a card for an archaeon (free choice of organism) to replace a question on archaea during the exam. These cards were not discussed in class and only evaluated as part of the exam, with the assessment based on the completeness of the information supplied on each card. The same criteria applied to the cards prepared for bacteria were used, taking into account that for many archaea morphology or physiology are still unknown. These cards were not used for the game as they were prepared after the end of the semester.

### Directed gameplay

Once all the cards were completed, students were encouraged to play a first session based on a game with pre-determined dynamics (Table 2; Supplementary Data 6). Based on this basic game, the students proposed two new variants of game dynamics and rules. In addition, two groups developed their own rules (Supplementary Data 6). For instance, students proposed gameplays based on the popular games ‘UNO’ or ‘Guess Who’. In the first case, the students used links between the characters (e.g., Gram-straining, classification, habitat) to place the cards on top of each other. For the second game, the player had to guess the character by asking the other players questions. In all cases, the focus of the games was the learning of the specific vocabulary and individual capacities of the characters.

### A Feast for Micro-organism

**Fig. 4.**
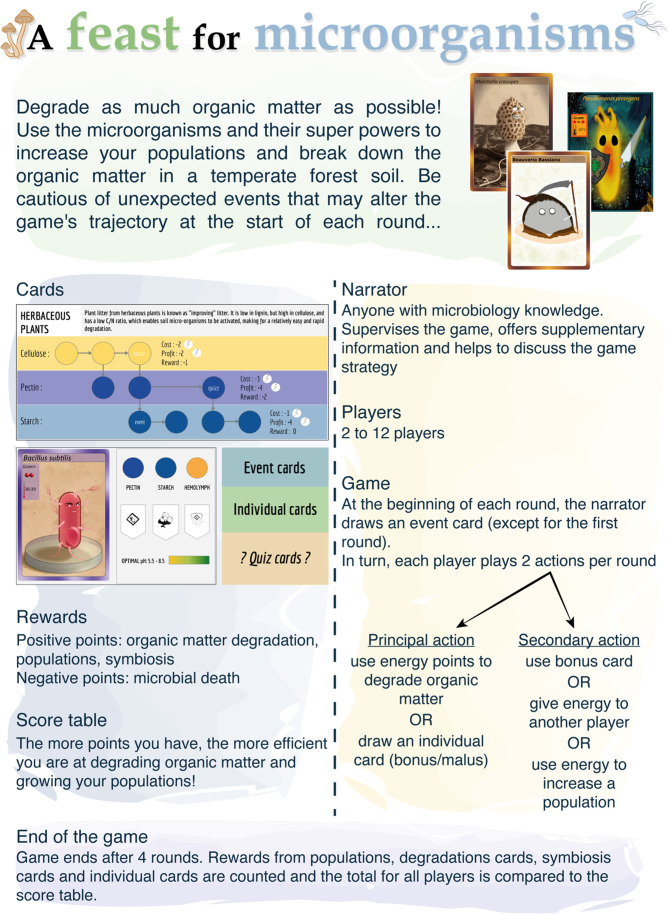
A Feast for Micro-organisms. Turn-based strategy game with the aim of degrading as much organic matter (degradation cards) as possible in four rounds. Twelve cards with different characters with specific powers are available and accumulate rewards. At the end of the game, the number of rewards indicates if the applied strategy was successful or not. Event cards induce a perturbation at the beginning of each round, while individual cards give a bonus/penalty. Quiz cards serve to improve students’ microbiology knowledge.

Using the cards made by the second-year students, the third-year students developed a cooperative game (‘A Feast for Micro-organisms’) that linked practical hypothesis validation to ecological functions. They selected, as the aim of their game, the degradation of the different organic matter sources available in the litter from a temperate forest (Fig. 4). This function was selected because it allowed them to also use knowledge acquired in other fields, such as soil sciences or functional ecology. The students selected 12 micro-organisms and tested their degradation capacities experimentally to confirm and complete the information found in the literature (Table 5). As this activity was part of an immersive module of problem-based learning, the students were given minimal guidance for the development of the experimental approach. They used literature research to identify experimental tests that could recapitulate key physiological functions associated with the ability of a micro-organism to participate to the degradation of organic matter. Considering the limited time available to perform the experiments (3 weeks), the students focused on the degradation of organic molecules found in plant litter (starch, pectin, lignin, cellulose and organic nitrogen). The combined literature search and experimental results were used to homogenize the content of the cards. However, only in some cases could the students compare the literature information with the experimental results (Table 5).

**Table 5.** Summary of the results of the degradation experiments

	**Degradation**				
	**Lignin**	**Cellulose**	**Starch**	**Pectin**	**Organic nitrogen**
*Bacillus subtilis*	Yes^+^	Yes	Yes	No	Yes
*Cupriavidus necator*	Yes^+^	Yes	nd	No^+^	Yes
*Pseudomonas protegens*	Yes^+^	Yes	nd	No	Yes^+^
*Pseudomonas putida*	Yes^+^	Yes	Yes	No^+^	Yes
*Streptomyces avermitillis*	Yes^+^	Yes	Yes	No^-^	Yes^-^
*Aspergillus niger*	No^-^	Yes^+^	Yes	No^-^	Yes^-^
*Beauveria bassiana*	No^+^	nd	Yes	No	Yes
*Coprinopsis cinerea*	No^-^	Yes	Yes	No	Yes
*Fusarium oxysporum*	No^+^	nd	Yes	No	Yes
*Morchella crassipes*	No^-^	Yes^+^	Yes	No	Yes
*Trametes versicolor*	Yes^+^	nd	Yes	No	Yes
*Trichoderma viride*	No	nd	Yes	No	Yes

Each experiment was done in triplicate. nd, Not determined (unclear experimental results); +, result corresponding to the literature information; −, result not corresponding to the literature information.

In addition, considering the high C/N ratio of litter, nitrogen fixation was also tested as a mechanism to supplement nitrogen to the degrading microbial community. However, the latter produced unreliable results that could not be verified in the time assigned for the experiments. With the help of these experimental results, the reverse sides of the character cards were homogenized, in order to indicate the degradation capacities of the organism, its superpowers, and optimal pH for functional activity.

For the purpose of the game, degradation boards, event cards, individual cards and quiz cards were developed (Fig. 4). The five degradation boards contained a brief description of the origin of the organic matter and its content in degradable products. The event cards had a positive or negative influence on the game, while the individual cards gave a bonus or penalty to the players. Energy coins were used to degrade the organic matter and to determine the growth of the character’s population. The game used a classical turn-taking strategy with energy coins and rewards, based on the degradation properties of the characters. Reward coins were received for successful organic matter degradation, population size increase, and the establishment of symbiosis. The total of rewards was calculated at the end the game to evaluate the success of the applied strategy for the degradation of the organic matter. In addition, a series of quiz cards were designed in order to reinforce the learning of microbiology concepts in a playful manner during the game. These cards could be answered by any player and corresponded to a multiple-choice question, with topics including the biotechnological use of specific microbes, the life cycle of specific fungi, or the physiological preferences of the organisms in the game. Upon a correct answer, the player obtained additional reward coins.

In order to evaluate the efficiency of the game as an educational tool, the students developed a survey about the overall appreciation of the game by the players (fellow third-year students; Supplementary Data 7). The game received an overall positive review, and the theme of the game (i.e., organic matter degradation) was said to be presented in a clear and interesting manner by all the participants. However, it was difficult for the players to clearly evaluate the effect of the game on learning and information retention.

## Discussion and perspectives

The aim of this project was to develop a game about bacteriology and mycology to help second- and third-year students to learn and consolidate the concepts studied during the lectures in a playful and stimulating manner. Second-year students developed character cards based on micro-organisms and their characteristics, while third-year students developed game rules to include ecological functions. The game therefore served two purposes: facilitating the learning of basic bacteriology and mycology concepts for the second-year students and consolidating the knowledge of multiple disciplines for the third-year students.

To achieve the learning objective for the second-year students, working on the cards was essential. However, some changes to the design of the cards could further improve their usefulness in the learning of certain basic concepts. For example, the students were able to discriminate Gram-positive and Gram-negative bacteria based on the coloration of the characters (violet *versus* pink). However, the cards did not provide information about the structure of the cell wall. The inclusion of a schematic representation of the cell wall would facilitate the learning of the structure and to reinforce the link between structure and Gram-staining. The same could be said for fungi, for which septate mycelium and clump junctions were incorporated in the designs of the characters; however, most students did not realize that the drawing represented the actual structure of the mycelium or its importance for taxonomy.

Although the second-year students were invited to develop new game dynamics, their knowledge level limited the dynamics proposed to the assimilation of the concepts and memorization of vocabulary. In contrast, by focusing on the role of micro-organisms in the degradation of organic matter, the third-year students were able to incorporate and reinforce knowledge acquired beyond concepts covered in microbiology. Those included aspects of soil science, botany and functional ecology. Additionally, as the third-year students developed the game as part of a problem-based learning module, they also developed skills in time management, experimental design, and game design.

Despite the overall positive rating of the cooperative game developed by the players (peers from the third year), the participants identified certain features, such as variable difficulty (highly difficult in the first turns, very easy in the final turns) and the absence of biological interactions, as aspects that should be considered for improvement in the game. In addition, increasing the population size (which was an action during the game) only boosted the number of rewards obtained, and did not have an impact on the degradation rate of the organic matter. Another feature that would be interesting to develop is symbiosis, which was only briefly covered in the game. Specific symbioses such as the one between rhizobia and the roots of leguminous plants could also open up the possibility of adding atmospheric nitrogen fixation as a key function, and the importance of the carbon/nitrogen ratio of the substrate in degradation success. Along the same lines, a physical game board would be an interesting addition to the game to mimic the heterogeneity of soil. Likewise, considering the role of viruses would be another aspect that could further enrich the game. Bacteriophages are known drivers of evolution and changes in function in bacterial populations [14], and also respond to the physical dynamics of heterogeneous environments such as soils [15]. Mycoviruses (viruses of fungi) are also reported to be ubiquitous throughout the fungal kingdom, and their importance in fungal biology is starting to be revealed, mainly through the analysis of fungal transcriptomes [16]. This opens up a new dimension in which elements of bioinformatic prediction of viral associations could also be included in future iterations of the game, or extensions of the game to other topics (e.g., bioinformatic data analysis).

The third-year students were also able to design *in vitro* experiments in order to confirm and complete the information found in the literature. This can turn laboratory experiments (that can be seen as an unrelated assignment) into a truly integrated learning experience, as discussed by the American Society for Microbiology Task Force on Curriculum Guidelines [5]. The basic degradation experiments performed by the students can be combined with additional ones in order to determine, for instance, the interactions (positive and negative) occurring between different characters in confrontation assays. This could include experiments such as plaque counting or phage isolation, or as indicated previously, the bioinformatic prediction of metabolisms or interactions, which can enrich the overall experimental elements of the activity.

In terms of evaluating the effectiveness of the development of the cards as a teaching tool, the second-year students were given the option to participate in the activity or not, with the goal of creating a no-game control group. However, in the end, all the students decided to participate, limiting our ability to evaluate the impact of the activity on the learning outcome, as done in other studies [6]. Nevertheless, the utility of the game could be observed indirectly by exam questions in which the knowledge in the cards had to be combined with theoretical content in lectures. The students only had the information on the cards, but in a large majority of cases were able to use the two sources of information (i.e., cards and lectures) to correctly answer the questions. One example involved the use of an agar deep tube (studied during the lectures) to represent the oxygen requirements of multiple organisms represented in the cards. This shows the connection made between the vocabulary and theory developed in the lectures and the concrete representation of the concepts made in the cards. Moreover, by structuring the laboratory experiments in a way that they connect directly with the game and its outcome, the principles and concepts of the lectures are connected not only with the laboratory, but also with real-world scenarios [5]. Finally, combining multiple approaches, including the use of video games for general or specific topics [17, 18], and reinforcing not only the link between micro-organisms and disease but including aspects such as the positive role of micro-organisms in the functioning of the biosphere, would result in more enriching and impactful learning of microbiology.

## Supplementary Data

Supplementary material 1
