# Supplementary material for: Gamification as a tool to teach key concepts in microbiology to bachelor-level students in biology: a case study using microbial interactions and soil functioning
Source: Access Microbiol. 2024 Feb 6;6(2):000699.v3. doi: 10.1099/acmi.0.000699.v3 (PMC10928398; doi:10.1099/acmi.0.000699.v3)
Supplement: Supplementary material 1 [file acmi-6-699.v3-s001.pdf]

Supplementary information

Supplementary Data 1. Categories for the concepts in English and in French. *As the official language for the bachelor students is French the cards were developed in French. A brief description of the expected information is given for each category.*

Bacteria

**English**	**Français**	**Description**
Morphology	Forme	Bacillus, coccus, etc.
Gram staining	Coloration Gram	Staining result
Oxygen requirement	Oxygène	Oxygen requirement (aerobic, anaerobic, etc.)
Optimal temperature	Température	Optimal growth temperature
Optimal ph	Ph	Optimal growth pH
Carbon source	Carbone/Nutrition	Hetero-, autotrophy, special nutrient requirements
Motility	Mobilité	Flagella, pili, etc.
Ecology	Ecologie	Lifestyle
N_2 fixation	Fixation azote (super pouvoir)	Fixation of atmospheric nitrogen
Antibiotics	Antibiotiques	Production or resistance
Biocontrol agent	Agent de biocontrôle (super pouvoir)	Use as a biocontrol agent
Sporulation	Survie	Formation of resistance structures
Model organism	Organisme modèle (super pouvoir)	Model organism in microbiology
Symbiose	Relation symbiotique	Symbiosis or endosymbiosis with a plant or another organism

Fungi

**English**	**Français**	**Description**
Phylum	Phylum	Ascomycota, Basidiomycota, ...
Vegetative structure	Forme végétative	Septate or non-septate mycelium, yeast, ...
Asexual reproduction	Reproduction asexuelle	Formation of conidia
Sexual reproduction	Reproduction sexuelle	Fructification, sexual spores (asci, basidia)
Ecological category	Catégorie écologique	Saprophyte, parasite, pathogen
Life style	Forme de vie	How we see it in the nature (mould, fruiting bodies, etc.)
Ecological role	Rôle écologique	Decomposition, population regulation, degradation of dead wood, etc.
Biocontrol agent	Agent de biocontrôle (autre)	Use as a biocontrol agent
Antibiotics	Antibiotiques (autre)	Production and resistance

CONFIDENTIAL

28 ***Supplementary Data 2. Media composition.***

**Starch medium**<br>Culture medium containing a basic mineral medium and starch as the sole carbon source. Amylolytic activity is detected by the addition of lugol (iodinated potassium iodide solution), which colours the starch brownish-purple. An absence of colouration indicates starch degradation.	K_2HPO_4<br>NaCl<br>SL6 solution<br>Soil extract<br>Soluble starch<br>NH_4NO_3<br>Agar<br>Deionised H_2O	0,2 g<br>0,1 g<br>1 mL<br>10 mL<br>1,5 g<br>1 g<br>15 g<br>ad 1L
**CMC medium**<br>Culture medium containing a basic mineral medium and carboxymethyl-cellulose (CMC) as the sole carbon source. The cellulolytic activity of microorganisms growing on this medium was detected by staining with Congo red (0.1%) for 40 minutes followed by washing with 1M NaCl.	K_2HPO_4<br>$(NH_4)_2SO_4$<br>$MgSO_4 \cdot 7H_2O$<br>NaCl<br>Carboxymethyl-cellulose sodium<br>Agar<br>Deionised H_2O<br>pH 5	1 g<br>1 g<br>0,5 g<br>0,5 g<br>5 g<br>20 g<br>ad 1 L
**RBBR medium**<br>Yeast extract-based culture medium with added dye: Remazol Brilliant Blue. The change from blue to transparent indicates ligninolytic activity.	NaCl<br>Yeast extract<br>MES buffer<br>Remazol brilliant blue<br>Agar<br>Deionised H_2O<br>pH 5<br><br>Sterilise the Remazol Brilliant Blue by filtration at 0.2µm and add to the medium after autoclaving.	1 g<br>0,1 g<br>1,95 g<br>0,5 g<br>20 g<br>ad 1 L
**Skimmed Milk medium**	Skimmed milk in powder	10 g

Culture medium containing malt extract and milk powder, which contains casein as a source of organic nitrogen. The hydrolysis of the casein is visible thanks to the translucent halo around the colonies.	Yeast extract<br>Agar<br>Deionised H_2O	12 g<br>15 g<br>1 L
**Pectin medium**<br>Culture medium containing pectin. The liquefaction of the of the medium indicate the degradation of the pectin.<br>Prepare the two media separately and mix them after autoclaving	$CaCl_2$<br>Agar<br>Deionised H_2O<br><br>Pectin<br>Ethanol<br>Glucose<br>K_2HPO_4<br>KH_2PO_4<br>$CaCl_2$<br>$MgSO_4 \cdot 7H_2O$<br>$Na_2MoO4 \cdot 2H_2O$<br>NH_4NO_3<br>$FeCl_3$<br>$CaCO_3$<br>Agar<br>Deionised H_2O<br>pH 8	2,5 g<br>7,5 g<br>500 ml<br><br>8 g<br>15 ml<br>20 g<br>0,05 g<br>0,15 g<br>0,01 g<br>0,2 g<br>0,002 g<br>1 g<br>0,01 g<br>1 g<br>15 g<br>ad 500 ml

CONFIDENTIAL

Supplementary Data 3. Table with the information compiled by the students for the bacteria.

	Morphology	Gram	Temperature	pH	Oxygen	Carbon	Habitat/Distribution	Nutrition	Ecology	Symbiose	Super power	Smell	Antibiotics	Sporulation	Motility
Ammoniphilus oxalaticus	bacillus with cilia, flagella and organelles	variable Gram	28-30°C	6,8-9,5	aerobic	obligate oxalotroph	oxalate-rich soil, decomposing wood	oxalate						endospores	peritrichous flagella
Anabaena azollae	cyanobacteria, filamentous assembly of vegetative cells and heterocysts	Gram -	18-30°C, mesophile, tropical weather	6,5-7,5	facultative aerobic	photoautotrophic	water			symbiosis with Azolla, an aquatic fern, organic carbon for atmospheric nitrogen fixation	atmospheric nitrogen fixation, can produce toxins, biofertiliser in rice paddies			akinetes	gliding
Bacillus subtilis	bacillus with peritrichous cilia/flagellae	Gram +	28-40°C	5,5-8,5	optional aerobic	heterotrophic	ubiquitous				model for the study of spore-forming bacteria, antagonist of pathogenic fungi		fungicidal activity	endospores	peritrichous ciliate (flagella)
***Bdellovibrio* sp.**	comma-shaped bacillus (vibrio) with polar-sheathed flagellum	Gram -	28-30°C	6,0-8	compulsory aerobic	heterotroph	aquatic environments		predator or parasite of Gram - bacteria		induces resistance to streptomycin in its host			bdelloplast	polar flagellum
***Borellia* sp.**	spirochete with endoflagellum		33-35°C	6,0-8	aerobic	heterotroph					uses tick saliva to avoid the immune system		resistance by division into small pieces		
***Chlamydia* sp.**	coccus	Gram -	35-39°C	8	aerobic		eukaryotic cells		intracellular parasite		resistant to antibiotics				non-motile
***Clostridium* sp.**	bacilli with peritrichous flagella	Gram +	20-50°C	5,5-8	anaerobic	heterotroph			saprophyte		certain strains are pathogenic, therapeutic agents against cancer			endospores	flagella
Cupriavidus necator	bacilli with peritrichous flagella	Gram -	22-30°C, mesophile	6,8-7,5	aerobic	heterotrophic or autotrophic in some cases	soil		sometimes a predator of bacteria and fungi		resistant to heavy metals, used in the production of bioplastics, biofuels or microbial fuel cells for electricity generation				flagella
Helicobacter pylori	spiral-shaped,flagellated	Gram -	35-37°C	3,5-8,6	micro-aerophilic	heterotroph	stomach		asymptomatic pathogen		production of urease, resistant to acid				flagella
***Heliobacterium* sp.**	bacilli with flagella	Gram +	25-30°C	7	anaerobicstrict	anoxygenic photoheterotrophic	aquatic environments waterlogged soils							endospores	flagella
Mycoavidus cysteinexigens	bacillus	Gram -	4-35°C	7	strict aerobic	heterotroph	cells of *Mortierella elongata*	needs cysteine for growth	endosymbiont	symbiosis with *M. elongata* , food against protection against nematodes					
***Mycoccocus* sp.**	bacilli and cocci	Gram -	14-40°C	5,2-8,5	obligate aerobic	heterotroph	soil		saprophyte, predator					myxospores	pili, ability to slide
Pseudomonas protegens	bacilli with flagella	Gram -	25-35°C, mésophile	7	strict aerobic	heterotroph	soil, plant roots				plant protection against various phytopathogenic fungi, fluorescent		antimicrobial production		flagella (1-3)
Pseudomonas putida	bacillus with frimbria, pili and polar lophotrichous flagella	Gram -	25-30°C	7,0-8,0	strict aerobic	heterotroph	soil, water, human ...		saprophyte, opportunistic pathogen		hydrocarbon degradation		resistant to certain antibiotics		polar lophotrich flagella
***Rhizobium* sp.**	bacilli with monopolar or peritrichous flagella	Gram -	25-30°C	6,5-7,5	aerobic	heterotroph	soil, plant roots		symbiont of leguminous plants	symbiosis with legumes, food against nitrogen compounds	nitrogen fixation, antibiotic resistance				peritrichous or monopolar flagella
***Rhodobacter* sp.**	ovoid or bacilli	Gram -		6,0-9	aerobic or anaerobic	photoautotrophic or photolithotrophic	water				nitrogen fixation				flagellum for some species

CONFIDENTIAL

***Streptomyces* sp.**	filamentous, mycelial growth, aerial hyphae	Gram +	15-40°C, mesophile	6,5-8	aerobic	heterotroph	soil	saprophyte	symbiosis with various plants, food versus antibiotics	resistance to NO	smell of the earth	resistant to spiramycin, producer of streptomycin, and antibiotics	spores
***Wolbachia* sp.**	bacilli and cocci	Gram -	host temperature	host pH			insects, nematodes	endosymbiont or parasite Intracellular	endosymbiont of nematodes	influences reproduction to favour the reproduction of infected individuals			

CONFIDENTIAL

Supplementary Data 4. Table with the information compiled by the students for the fungi.

	Phylum	Vegetative structure	Asexual reproduction	Sexual reproduction	Habitat	Ecological category	Life style	Ecological role	Other
Aspergillus niger	Ascomycota	partitioned mycelium	round conidia formed on a vesicle carried by the conidiophore (pompom)	not described	ubiquitous, soil and decomposing organic matter	saprophyte, sometimes animal pathogen	saprophyte on fresh organic matter	degradation of organic matter -> return of nutrients to the soil	cosmopolitan
Batrachochytrium dendrobatidis	Chytridiomycota	holocarpic with zoosporangium	production of zoospores	not described	amphibian skin, aquatic environment	parasite	parasitic on amphibians	regulates amphibian populations	causes chytridiomycosis
Beauveria bassiana	Ascomycota	partitioned mycelium	blastospore-type conidia	perithecia with asci grouped in stromata, called Cordyceps in the sexual phase	insects, soil	parasite and saprophyte	enthomopathogenic (for the parasitic stage)	regulates insect populations	used as a biological control agent against crop pests
Candida albicans	Ascomycota	yeast or mycelium	blastospore-type conidia	formation of asci and ascospores without fructification. Probably heterothallic bipolar	mucous membranes, human commensal fungi	saprophyte, opportunistic pathogen	if parasitic/pathogenic: symptoms of disease such as thrush	part of a healthy human microbiota	vegetative form dependent on pH, facultative anaerobic, can become pathogenic under stress
Coprinopsis cinerea	Basidiomycota	mycelium with clamp connections	fragmentation of the mycelium (arthrospores)	basidiospores on basidia assembled on a lamellar hymenium present on the lower surface of the fruiting body. Dispersal by autolysis of fungal tissues. Tetrapolar heterothallic system.	manure, dung	saprophyte	hairy coprin, coprophagous saprophyte	breaks down organic matter with a low C:N ratio (= rich in N)	produces antimicrobial and anti-nematode compounds
Entomophthora muscae	Zoopagomycota (informal Zygomycota)	coenocytic mycelium	blastospore-type conidia	not formally described, but probable on the basis of population genetic data	flies	parasite	fly parasite	regulates fly populations	modifies the behaviour of infected flies to promote its spread.
Fusarium oxysporum	Ascomycota	partitioned mycelium	formation of macro and microconidia and chlamydospores	not described	host plant, soil	plant parasite, symbiont, saprophyte	variable	Very varied ecology. Restores nutrients to the soil (saprophyte), improves plant growth (symbiont) or regulates plant populations (pathogen).	High temperatures, dry soil and neutral pH. Can solubilise gold!
***Malassezia* sp.**	Basidiomycota	yeast, mycelium	conidia / multiplication in yeast form	not formally described, but MAT locus genes detected	epidermis, digestive system, human commensal, ubiquitous (e.g. environmental surfaces)	saprophyte, commensal, opportunistic pathogen	no particular form	decomposer of oragnic matter -> returning nutrients to the soil	can become pathogenic if immunodeficient
Morchella crassipes	Ascomycota	mycelium	conidia	asci and ascospores within a hymenium organised into several apothecia grouped on a fruiting body	soil	saprophyte and mutual symbiont	morel, ectomycorrhiza	returning nutrients to the soil (saprophytes), improving plant growth (mycorrhizae)	Heat shock necessary for fruit formation. Highly prized edible!
***Neocallimastix* sp.**	Neocallimastigomycota	Monocentric eucarpic thallus, filamentous rhizoids	flagellate zoospores	not described	rumen	mutual symbiont	rumen symbiont	participates in the turnover of cellulose ingested by herbivores.	obligatory anaerobic, no mitochondria
Paxillus involutus	Basidiomycota	mycelium	fragmentation of the mycelium	basidiospores on basidia clustered on a lamellar hymenium present on the lower surface of the fruiting body.	soil, roots	symbiont	ectomycorrhiza	improves plant growth and therefore primary production	model organism for the study of ectomycorrhizal symbiosis

Rhizophagus irregularis	Mucoromycota (Glomeromycotina)	coenocytic mycelium	production of multinucleate spores (actually the sporangium) larger than the hyphae.	not described, but MAT locus detected	soil	plant symbiont	arbuscular endomycorrhiza	improved plant growth and therefore primary production via phosphorus acquisition, protection, increased surface area for absorption	obligatory biotroph
Rhizopus oligosporus	Zygomycota	coenocytic mycelium, multinucleate hyphae	formation of a sporangium and sporangiospores	two sexually differentiated mycelia (heterothalloque biploar system) and formation of zygosporangium and zygospores zygospores.	domesticated for use in tempeh production	saprophyte	no particular form	degradation of organic matter -> return of nutrients to the soil	production of antimicrobial metabolites, used in tempeh production, lipolytic and proteolytic activity
Trametes versicolor	Basidiomycota	mycelium in wood, with clamp connections	chlamydospores, fragmentation of the mycelium	basidiocarps in the shape of a tuille or rosette (annual fruiting bodies). Hyemium poré with basidia and basidiospores	dead hardwood trunks	saprophyte, white rot	rosettes on trees with alternating lines of colour, white wood rot	decomposition of dead wood, degradation of lignin, involvement in the carbon cycle and release of nutrients immobilised in the wood	Producers of active molecules such as antimicrobials, oxidative enzymes, used in prostate cancer research
Trichoderma viride	Ascomycota	partitioned mycelium	formation of conidia and chlamydospores	described by another name: *Hypocrea rufa* . Perithecial fructification with asci and ascospores	soil, plants, decomposing organic matter	saprophyte, symbiont, parasite	green mould	degradation of organic matter -> release of nutrients into the soil in the soil.	use as a biocontrol agent for fungal pathogens or to improve plant growth"
Ustilago maydis	Basidiomycota	yeast (monokaryon) and mycelium (dikaryon)	dicaryotic conidia for the mycelial phase and yeast phase multiplication.	plasmogamy of sexually differentiated monokaryotic yeast cells (tetrapolar heterothallic system), formation of dicaryotic teliospores on the host plant, release of basidispores via germination of the teliospore	soil, maize crops	saprophyte (yeast) or parasite (mycelium)	maize smut	release of nutrients for the decomposer part and regulation of plant populations for the parasite part	hot and dry climates

CONFIDENTIAL

Sup. Data 5. Pre-designed cards used in the game. Cards designed by Data-partner.ch

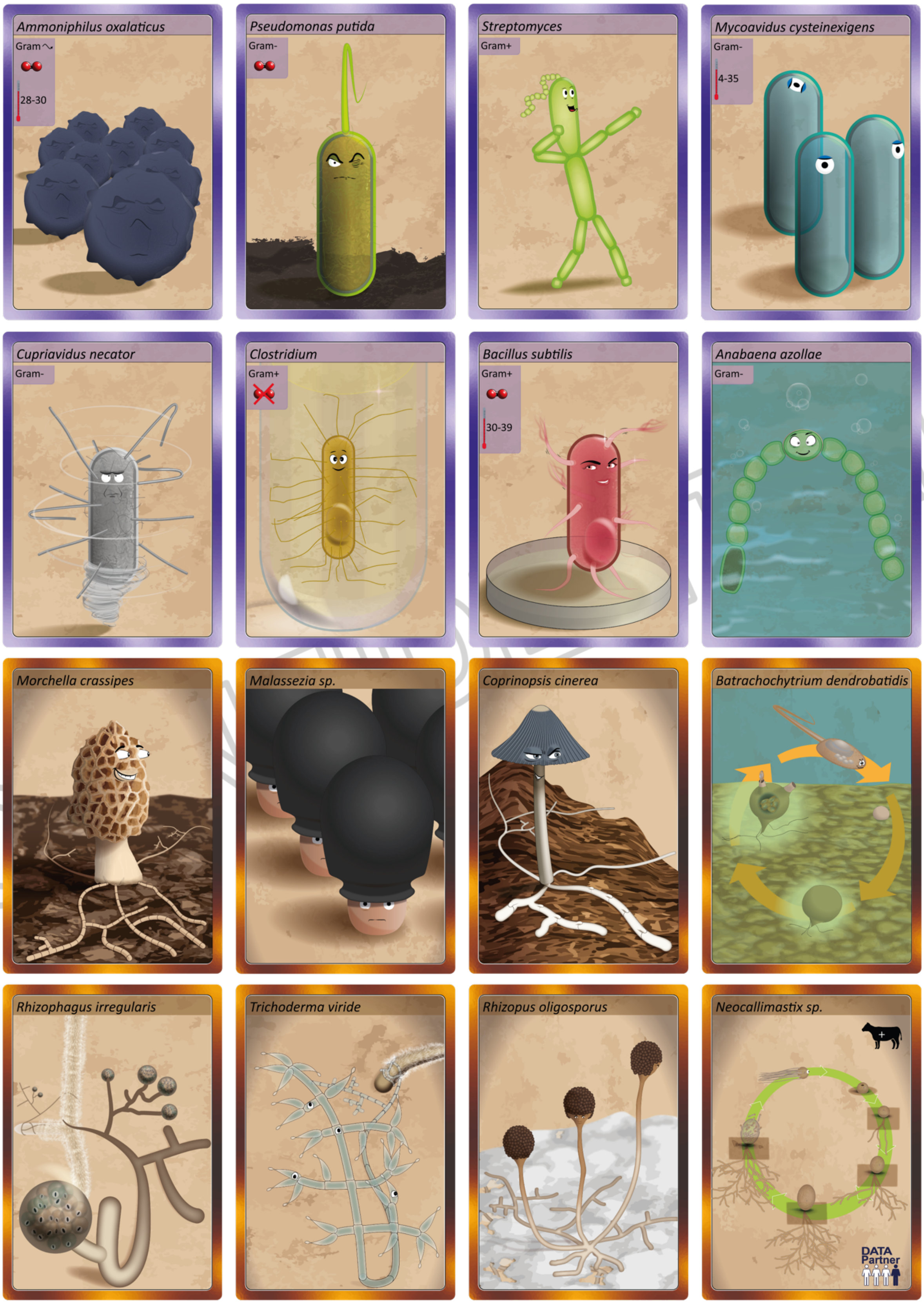

Supplementary Data 6- Game rules

#1 – Pre-defined game rules (Party-Invite) – 4-5 players

The goal of the game is to get rid of one's cards.

- Deal 4 to 5 cards (depending on the number of players) from the card's deck to each player.

- Place a room card in the middle.

- Taking turns, each player plays a card with a microorganism that meets the criteria to enter the room card.

- If the player makes a mistake, they take back their card and draw one as a penalty.

- If the player cannot play, they skip their turn.

- After each turn, a new room card is played.

Players are not allowed to look at the support table (Supplementary Data 3 and 4) before playing their card, but after playing it, they can check the Table. If the number of participants is too high, 1 to 2 players can act as referees and check the Tables.

#2 Variant proposed by the students

1) Distribute between 5 and 7 bacteria/fungus cards randomly to all players, depending on the number of players.

2) Place 6 "Room" cards in the middle.

3) Each player, in turn, places one of their cards onto one of the "Room" cards using their knowledge or the information written on their card without consulting the "information chart."

4) If a player doesn't know the characteristics of their bacteria/fungus card, they can bluff and place it on a "Room" card that doesn't actually match their card. However, any player can challenge the affiliation of a card to a house. In this case, verification is done using the players' knowledge and the provided information chart.

5) If a player challenges the placement, but the card was actually affiliated with the correct house, the challenger takes one card from the hand of the player they challenged. If a player challenges and the card was placed in a "Room" not related to their card, they, in turn, take one card from the hand of the player they challenged.

6) The first one to have no cards left wins.

CONFIDENTIAL

#3 – Variant proposed by the students

Take four “Room” cards. Each player in turns draws 1 card per turn and tries to place it on the “Room”; if unable to place the card, they keep it in hand.

When 3 cards are placed on a “Room”, the “Room” is full and it is removed and replaced by a new one.

For the *Rooms” hating, loving, and keeping Fungi at bay, one can place an organism card and a host card, and if a host card is placed by one person, others can also benefit from it. In the end, the player with the fewest cards in hand wins.

Note for improvement: motility and to cook “Rooms” could be added to place organisms that are known to be motile or can be used in cooking.

#4 - UNO

The principle is the same as for the traditional “*Uno*” game: the goal is to get rid of all your cards.

Shuffle all the cards and deal 4 to 7 cards to each player, depending on the number of players. Form a draw pile with the remaining cards.

Flip a card to create the base card. On top of it, players must place a card with a common feature (for example, a Gram+ bacterium can be placed on another Gram+ bacterium; a basidiomycete on another basidiomycete; a symbiont on another symbiont, etc.)

The room cards from the Party-invite game can be included. Those are often easier to play or can be used as the equivalent of the "change color" card, but there must be a connection with the last played card.

Variant: Form two groups and have them compete; the first group in which all players have played their cards wins.

Normal Uno rules apply: if a player forgets to say Uno when they have only one card left, they must draw 2 cards. The first player to get rid of all their cards as quickly as possible wins. In addition, to enhance the gameplay, it would be nice to include "+1 draw," "+2 draw," and "change direction" cards, similar to the traditional Uno.

CONFIDENTIAL

#5 – Guess who

We're playing “Guess who”! Each person is playing a character they do not know. Their goal is to figure out what organism they are by asking questions (e.g., am I a fungus? Am I Gram-positive, etc.).

The other players can only respond with yes or no (being careful not to give away too much information with comments).

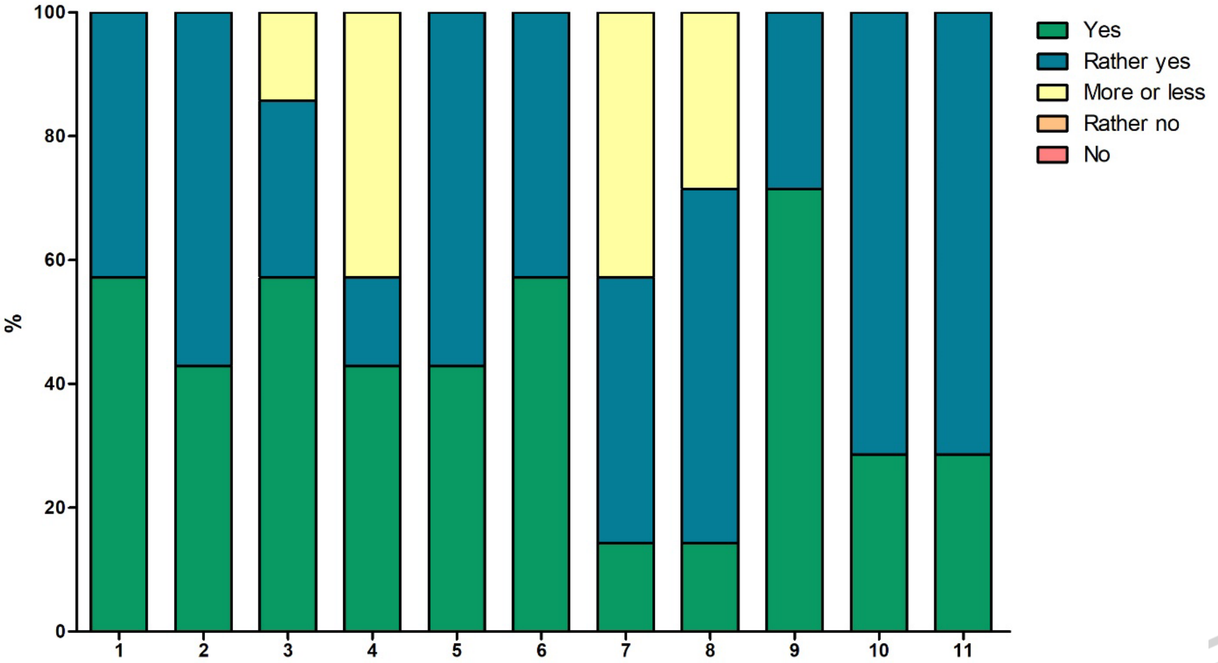

1. Overall, the game was enjoyed
2. The infographics and information were clear
3. The number of players was appropriate
4. The number of rounds was suitable
5. The amount of information allowed a good clarity and fluency in the game
6. The difficulty level of the information was adapted to your knowledge
7. I learned and was able to apply new knowledge
8. Did the game help to consolidate the concepts already covered in class?
9. The theme of organic matter degradation in the soil was addressed in a clear and interesting manner
10. The theme of symbiosis was addressed in a clear and interesting manner
11. The diversity and importance of the roles of microorganisms in the soil were addressed in a clear and interesting manner

Supplementary Data 7. Pedagogical appreciation survey. Students were invited to fill a survey after playing the game, in order to evaluate the efficiency of the game as an educational tool for the bachelor students. N=7.
